# Supplementary material for: Phosphatidylcholine Transfer Protein OsPCTP Interacts with Ascorbate Peroxidase OsAPX8 to Regulate Bacterial Blight Resistance in Rice
Source: Int J Mol Sci. 2024 Oct 26;25(21):11503. doi: 10.3390/ijms252111503 (PMC11546617; doi:10.3390/ijms252111503)
Supplement: Supplementary file 1 [file ijms-25-11503-s001.zip › Figure S1.pdf]

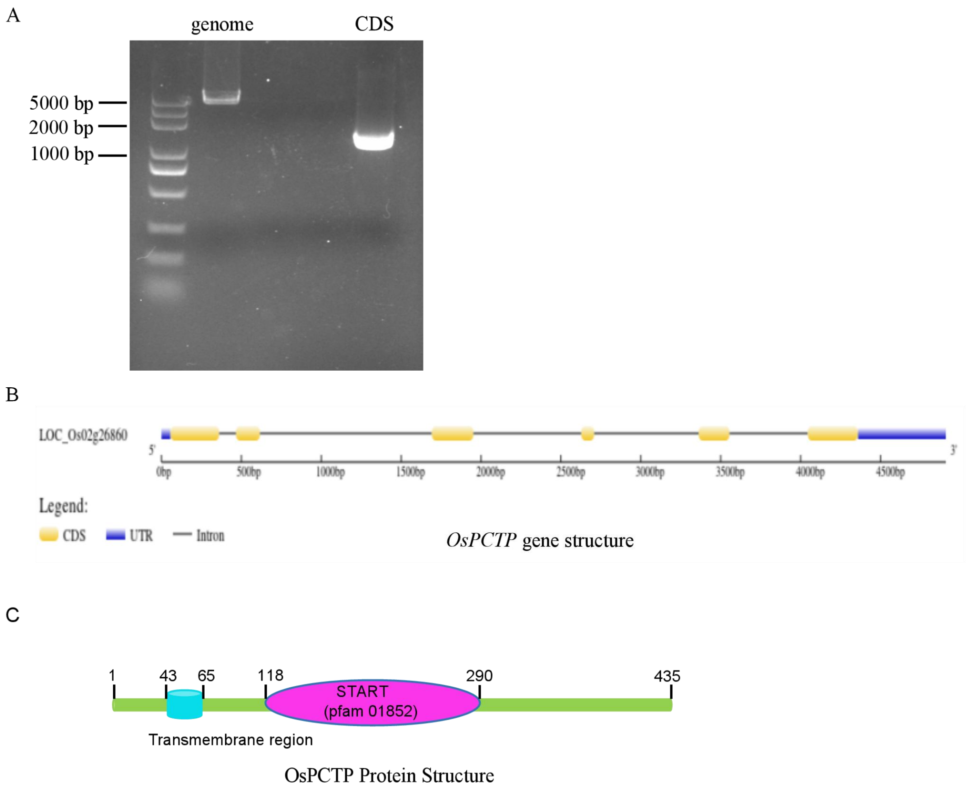

Figure S1. Gene and protein structure of *OsPCTP*. (A) The isolation of the genome and CDS sequences of *OsPCTP* from a rice leaf. (B) Schematic gene structure of *OsPCTP* in rice genome. (C) The protein structure domains in OsPCTP predicted with SMART database.
