# Supplementary material for: Phosphatidylcholine Transfer Protein OsPCTP Interacts with Ascorbate Peroxidase OsAPX8 to Regulate Bacterial Blight Resistance in Rice
Source: Int J Mol Sci. 2024 Oct 26;25(21):11503. doi: 10.3390/ijms252111503 (PMC11546617; doi:10.3390/ijms252111503)
Supplement: Supplementary file 1 [file ijms-25-11503-s001.zip › Figure S2.pdf]

A

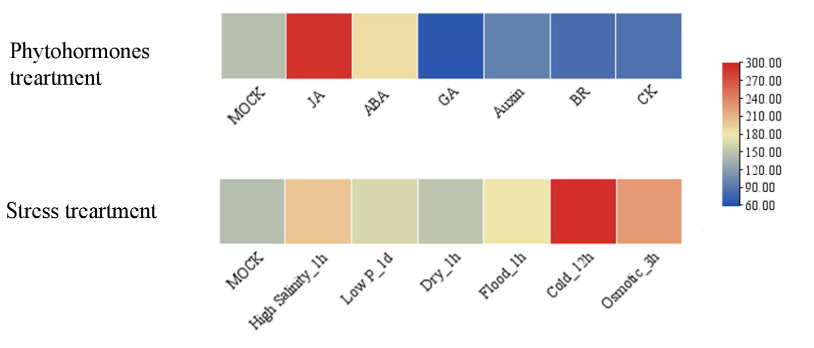

B

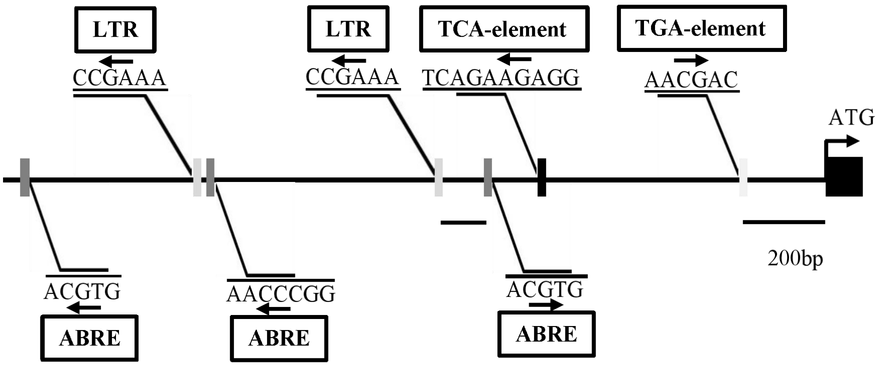

ABRE: abscisic acid responsive element; **TCA-element:** salicylic acid responsive element;
LTR: low-temperature responsiveness; **TGA-element:** auxin-responsive element;

Figure S2. *OsPCTP* may be induced by stress and hormome. (A) Heatmap of the expression of the *OsPCTP* in stress and phytohormones treartment; (B) Cis-elements analysis of *OsPCTP* promoter with PlantCARE database (http://bioinformatics.psb.ugent.be/webtools/plantcare/html/).
