# Supplementary material for: Phosphatidylcholine Transfer Protein OsPCTP Interacts with Ascorbate Peroxidase OsAPX8 to Regulate Bacterial Blight Resistance in Rice
Source: Int J Mol Sci. 2024 Oct 26;25(21):11503. doi: 10.3390/ijms252111503 (PMC11546617; doi:10.3390/ijms252111503)
Supplement: Supplementary file 1 [file ijms-25-11503-s001.zip › Table S1.pdf]

Table S1. Primers used for vector construction and Quantitative RT-PCR

Gene	Forward primer (5'-3')	Reverse primer (5'-3')
OsPCTP-OE	CTGATTAACAGGGATCCCCCATGGCGGGGGAGACGGATT	TCGAGACTAGTGGTACCCCCTTCCAAGTGCCTGGGTTCAGT
P1306-APX8	ACGGGGGACGAGCTCGGTACCATGGCGGAGCGCATCGCC	TTGGTCGACTCTAGAGGATCCGCTCCCGAGCAGAGACGTCA
PCTPF-nLUC	ACGGGGGACGAGCTCGGTACCATGGCGGGGGAGACGGATT	CGCGTACGAGATCTGGTCGACTTCCAAGTGCCTGGGTTCAGT
cLUC-APX8	TACGCGTCCCGGGGCGGTACCATGGCGGAGCGCATCGCC	AGTAGTCGATTTGTTGGATCCTCAGCTCCCGAGCAGAGACG
AD-APX8	GCCATGGAGGCCAGTGAATTCATGGCGGAGCGCATCGCC	ATGCCCACCCGGGTGGAATTCTCAGCTCCCGAGCAGAGACG
BD-PCTP	ATGGCCATGGAGGCCGAATTCATGGCGGGGGAGACGGATT	TCGACGGATCCCCGGGAATTCCTATTCCAAGTGCCTGGGTTCA
qRT-APX8	ATGCTAAACTGAGCGACCTT	GCACCGTTGTTGTCATCC
qRT-PR1b	CAAAACTCCCCGCAGGACTA	GAGGTTCTCGCCAAGGTTGT
qRT-PR1a	AAGCTGGAGCACTCGGACT	ACACCACCTGCGTGTAGTG
qRT-PAL1	AGCACATCTTGGAGGGAAGCT	GCGCGGATAACCTCAATTTG
qRT-PR10	CCCTGCCGAATACGCCTAA	CTCAAACGCCACGAGAATTTG
qRT-PCTP	CTTCATCACGCCAACAGC	TTCCAAGTGCCTGGGTTC
UBQ	AACCAGCTGAGGCCCAAGA	ACGATTGATTTAACCAGTCCATGA
